# Tricuspid Valve Repair Can Restore the Prognosis of Patients with Hypoplastic Left Heart Syndrome and Tricuspid Valve Regurgitation: A Meta-analysis

**DOI:** 10.1007/s00246-023-03256-0

**Published:** 2023-08-09

**Authors:** Matteo Ponzoni, Danila Azzolina, Luca Vedovelli, Dario Gregori, Vladimiro L. Vida, Massimo A. Padalino

**Affiliations:** 1 Pediatric and Congenital Cardiac Surgery Unit, Department of Cardiac, Thoracic Vascular Sciences and Public Health, University of Padua Medical School, Via Giustiniani 2, 35128 Padua, Italy; 2 Unit of Biostatistics, Epidemiology and Public Health, Department of Cardiac Thoracic and Vascular Sciences, University of Padua Medical School, Padua, Italy; 3 Department of Environmental and Preventive Sciences, University of Ferrara, Ferrara, Italy

**Keywords:** Hypoplastic left heart syndrome, Tricuspid valve regurgitation, Tricuspid valve repair, Review, Meta-analysis

## Abstract

To date, evidence supporting the efficacy of tricuspid valve (TV) repair in interrupting the progression of systemic right ventricular (RV) adverse remodeling in hypoplastic left heart syndrome (HLHS) is conflicting. We conducted a systematic review and meta-analysis of scientific literature to assess the impact of TV repair in effectively modifying the prognosis of patients with HLHS. We conducted a systematic review of PubMed, Web of Science, and Scopus databases. A random-effect meta-analysis was performed and transplant-free survival, freedom from TV regurgitation, and TV reoperation data were reconstructed using the published Kaplan–Meier curves. Nine studies were included, comprising 203 HLHS patients undergoing TV repair and 323 HLHS controls. The estimated transplant-free survival at 1, 5, and 10 years of follow-up was 75.5% [95% confidence interval (CI) = 67.6–84.3%], 63.6% [95% CI = 54.6–73.9%], and 61.9% [95% CI = 52.7–72.6%], respectively. Transplant-free survival was comparable to HLHS peers without TV regurgitation (p = 0.59). Five-year freedom from recurrence of TV regurgitation and freedom from TV reoperation was 57% [95% CI = 46.7–69.7%] and 63.6% [95% CI = 54.5–74.3%], respectively. Younger age and TV repair at the time of Norwood operation increased the risk of TV regurgitation recurrence and the need for TV reoperation. Our meta-analysis supports the efficacy of TV repair in favorably modifying the prognosis of patients with HLHS and TV regurgitation, reestablishing a medium-term transplant-free survival which is comparable to HLHS peers. However, durability of surgery and long-term fate of TV and RV performance are still unclear.

**Graphical Abstract:**

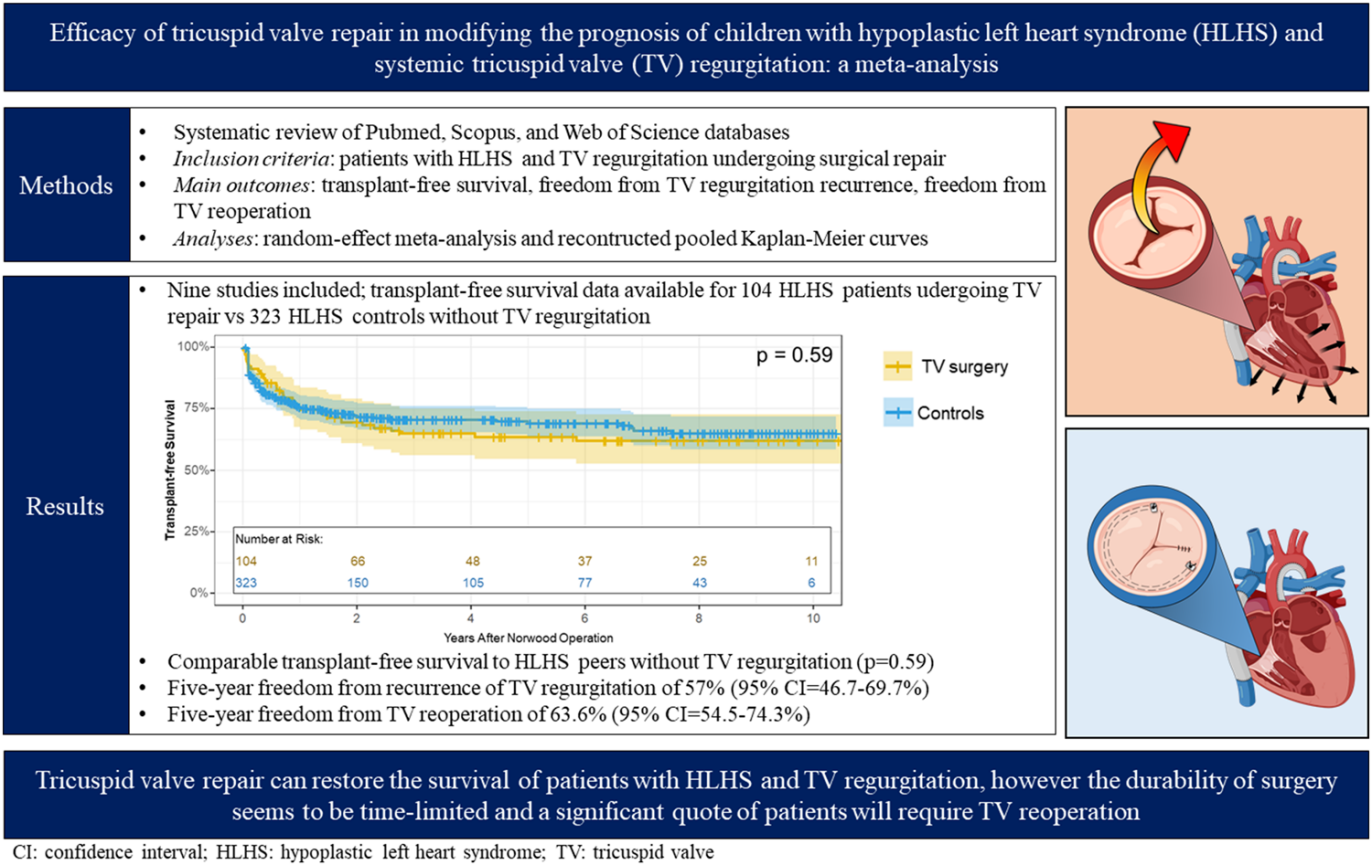

**Supplementary Information:**

The online version contains supplementary material available at 10.1007/s00246-023-03256-0.

## Introduction

The inclusion of the morphologically tricuspid valve (TV) into the systemic circulation by means of the Fontan palliation pathway triggers a premature deterioration of its competence [1–6]. Right ventricular (RV) dominance exposes univentricular patients to an increased risk of clinically significant atrioventricular valve regurgitation, which translates into poor early and long-term transplant-free survival [7–10]. Hypoplastic left heart syndrome (HLHS) represents the prototype of this condition [11] and 15–25% of affected patients are expected to require surgical management of TV regurgitation (TVR) during their palliation course [12–17].

**Fig. 1:**
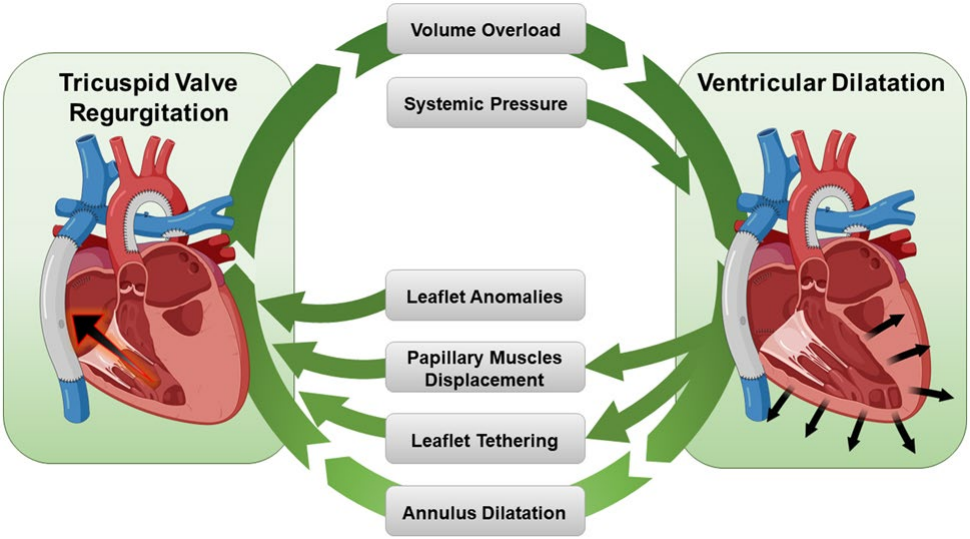
Pathophysiology of TVR in patients with HLHS undergoing single-ventricle palliation

The underlying pathophysiology of systemic TVR entails a vicious cycle where all the constituents of the TV apparatus, as well as the ventricular myocardium, are involved (Fig. 1). The systemic afterload imposes a pressure stress on the RV, which undergoes a remodeling process eventually leading to ventricular dilatation [18]. Papillary muscles displacement, leaflet tethering, and TV annulus enlargement contribute to the loss of coaptation [19, 20]. Also, intrinsic abnormalities of the TV leaflets can be present [15]. The development of TVR generates additional RV volume overload, which further deteriorates RV performance and aggravates TVR itself, finally resulting in failing Fontan circulation [21].

To date, the role of surgical TV repair in successfully interrupting this vicious cycle is still unclear. In fact, evidence supporting the efficacy of TV repair in reverting the (un)natural progression of RV adverse remodeling and preventing TVR recurrence in HLHS is conflicting [14, 20, 22–24]. This ultimately turns into a poorly predictable long-term outcome even for HLHS patients in whom a successful TV repair has been achieved [12, 14–16]. Moreover, the very small sample size of surgical cohorts dramatically limits the generalization of findings. We conducted a systematic review and meta-analysis of scientific literature to assess the impact of TV repair in effectively modifying the prognosis of patients with HLHS, the risk of TVR recurrence, and the need for reintervention.

## Materials and Methods

### Data Collection

A systematic review was conducted according to the PRISMA [25] and MOOSE [26] guidelines. This study was prospectively registered on the PROSPERO database (CRD42023396529). The PubMed, Web of Science, and Scopus databases were systematically searched in January 2023, by two authors (M.P. and M.A.P.). Any eligibility disagreement was resolved by discussion among all the authors and then agreement by consensus. Ethics approval and patient consent were obtained by each research group. Our institutional Ethics Review Board waived the need for ethics approval for the meta-analysis. Data available on request to the corresponding author.

### Inclusion Criteria

After duplicates removal, the manuscripts were firstly screened on the title and abstract and then underwent full-text revision, using the following inclusion criteria: (1) study population composed of patients affected by HLHS with TVR undergoing TV repair; (2) studies reporting survival, and/or TVR recurrence, and/or risk of reoperation for TVR displayed as Kaplan-Meier curves; (3) papers written in English after 1970.

### Exclusion Criteria

Excluded studies were the ones: (1) enrolling patients undergoing TV replacement as a primary surgical attempt for TVR; (2) enrolling univentricular patients without a specific anatomical diagnosis of HLHS; (3) series without surgical treatment of TVR; (4) case reports and series with less than five patients; (5) reviews and meta-analyses; (6) not full-text manuscripts.

### Data Extraction

Two authors (M.P. and M.A.P.) extracted data to a pre-set Excel abstraction form. Extracted data were: publication year, number of patients, number of controls, cohort period, age at study, follow-up period, gender, the timing of TV repair (classified as: during the Norwood procedure; interstage I; during the bidirectional Glenn; interstage II; during Fontan operation; after Fontan operation), the specific surgical technique for TV repair, transplant-free survival, patients at risk, freedom from TVR recurrence, freedom from reoperation for TVR, early (in-hospital) mortality, early (in-hospital) reoperation rate for TVR. When available, transplant free-survival and patients at risk were extracted also for control patients (i.e. those affected by HLHS without clinically significant TVR, not requiring TV repair) from the selected studies. Transplant-free survival curves were reconstructed by selecting only those studies in whom estimates were measured starting from the day of the Norwood operation, to allow for a comparison with controls.

### Quality Assessment

The risk of bias at the study level was assessed by two reviewers (M.P. and L.V.) using the Appraisal tool for Cross-Sectional Studies (AXIS) [27]. The AXIS 20-item tool assesses the quality of cross-sectional studies based on the following criteria: clarity of aims/objectives and target population; appropriate study design and sampling framework; justification for the sample size; measures taken to address non-responders and the potential for response bias; risk factors/outcome variables measured in the study; clarity of methods and statistical approach; appropriate result presentation, including internal consistency; justified discussion points and conclusion; discussion of limitations; and identification of ethical approval and any conflicts of interest. The scoring system conforms to a “yes”, “no”, or “do not know/comment” design. We classified the studies into four quality categories based on the number of “yes” answers for each of the 20 questions included in the AXIS tool [28]: “high” (> 15 positive answers), “medium” (between 10 and 15), “low” (between 5 and 9), and “very low” (< 5).

### Study Description

The study characteristics are presented descriptively as mean and standard deviation (SD) or median (interquartile range [IQR]) in the case of quantitative variables, depending on the data reported in the study, and as absolute and relative frequencies in the case of categorical variables.

### Meta-analysis

*Time to Event Endpoints.* The transplant-free survival and time to event data were reconstructed using the algorithm indicated by Guyot et al [29]. The global Log-rank test was reported on the plot. The pooled hazard ratios (HR) were calculated via the Cox regression model on the reconstructed individual patient data with their related confidence interval (CI). A frailty term has been included in the model to account for correlation within the data reconstructed in the same study. Survival curves were obtained with the Kaplan-Meier method. Outcomes were presented as pooled proportions for data synthesis.

### Other Endpoints

A random-effect meta-analysis has been carried out on the study outcomes. The heterogeneity is estimated from the studies’ intervention effects and standard errors included in the meta-analysis via Der Simonian and Laird Estimator [30]. The I² measure has been considered to quantify the heterogeneity. The measure expresses the percentage of between-study variability related to heterogeneity rather than chance [31]. The study-specific estimates with 95% CI have been reported representing the pooled meta-analytical estimate in a forest plot.

### Effect Modifiers

Univariable meta-regression models have been computed to assess whether the study characteristics may act as effect modifiers on the final meta-analysis estimate. Considered variables for meta-regression were: publication year, age at surgery, percentage of patients at Norwood stage, and follow-up time. Given the unavailability of patients’ gender data in most of the studies, this variable was not included in the meta-regression model.

### Publication Bias

The publication bias has been visually assessed by considering a Funnel plot representation. A funnel plot is a scatter plot of the study-specific effect sizes (log odds ratio or mean difference) against the standard error on the ordinate axis. When there is no publication bias, the data points in such a plot should form a roughly symmetrical, upside-down funnel. The symmetry has been also assessed by considering the linear regression test of the Egger Test for asymmetry in the funnel plot.

Computations were performed in R 4.0.1 [32] system with metaphor and IPDfromKM packages [33, 34].

## Results

**Fig. 2:**
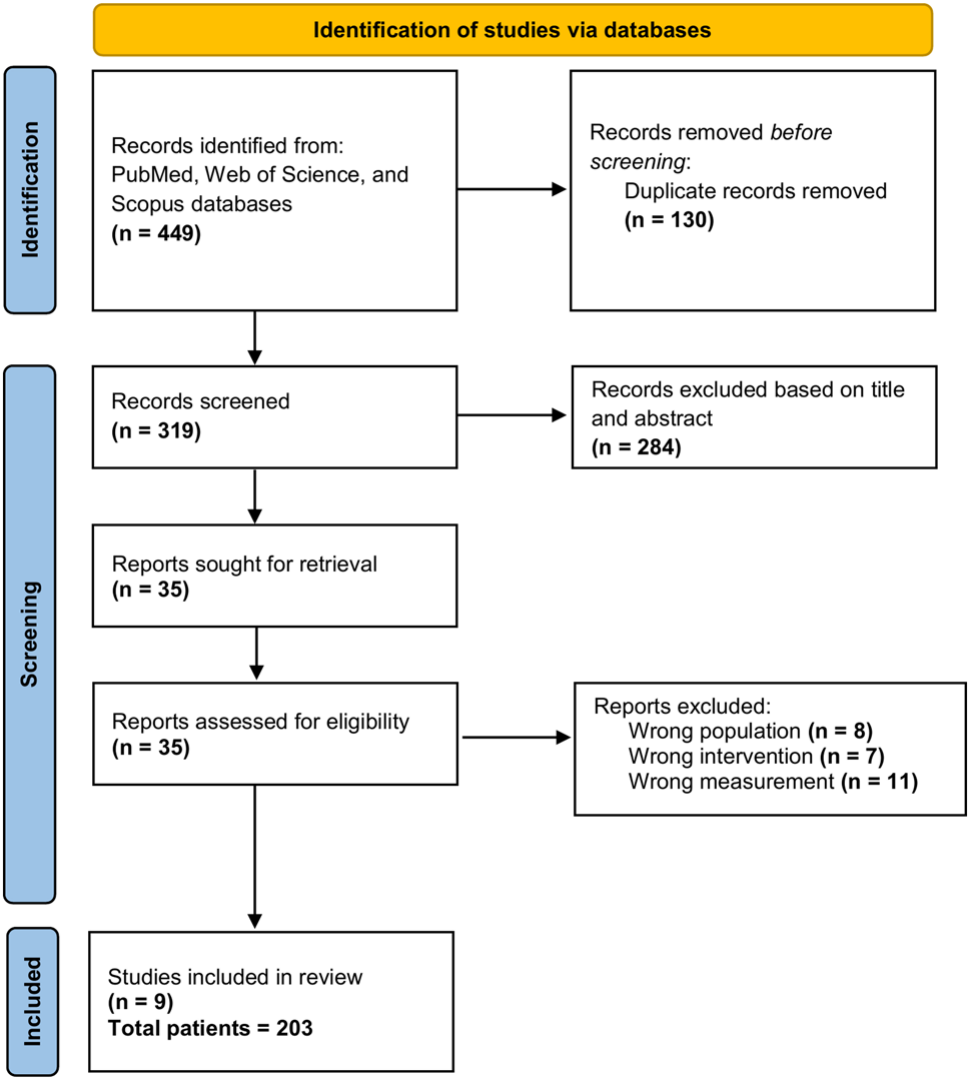
PRISMA 2020 flow diagram for new systematic reviews

**Fig. 3:**
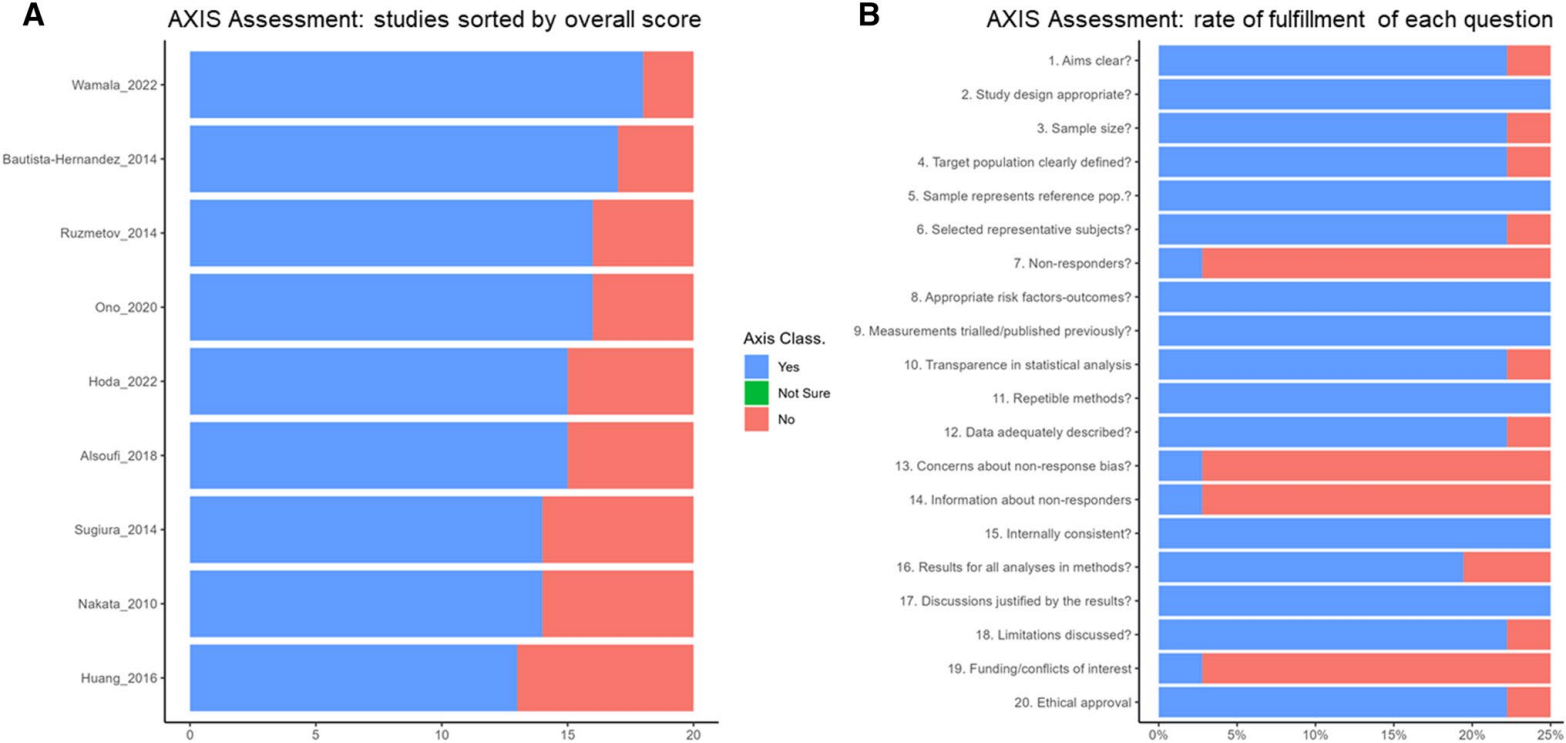
Quality assessment of manuscripts using AXIS tool: selected studies (n=9) sorted by overall quality (panel A) and rate of fulfilment of each quality item of AXIS tool across papers (panel B). Blue color indicates AXIS criteria fully satisfied; red color indicates AXIS criteria not satisfied; green color indicates AXIS criteria not evaluable

After the removal of duplicates, a total of 319 manuscripts were identified; full-text eligibility was assessed for 35 of them and, finally, 9 articles could be included (Fig. 2; Table 1) [12–16, 22, 35–37]. Figure 3 summarizes the quality assessment of selected reports using AXIS tool. Quality resulted in being high in 4 (44%) of studies and medium in 5 (56%). Quality assessment of each manuscript is provided in Supplemental Fig. 1.

### Patient Characteristics

We identified a total of 203 patients who underwent surgical repair of TVR across series. The median/mean age at TV repair ranged from 0.02 to 1.9 years (Table 1). The majority of operations occurred concomitantly to the scheduled palliation procedures: 50 (24.6%) at the time of Norwood operation, 9 (4.4%) during interstage I period, 79 (38.9%) at bidirectional cavo-pulmonary connection (bidirectional Glenn) surgery, 15 (7.4%) during interstage II period, 61 (30%) at Fontan operation, and 9 (4.4%) after Fontan completion (Supplemental Fig. 2). The median/mean follow-up ranged from 0.4 to 7.9 years.

**Table 1:** Selected studies (n = 9) and demographic characteristics of patients

Author	Year	Cohort Period	Cases	Controls	Age at TV repair (years)	Male	Follow-up (years)	Timing of TV repair (n)					
			(n)	(n)	median (IQR)/mean (SD)	(n)	median (IQR)/mean (SD)	At Norwood	Interstage I	At Glenn	Interstage II	At Fontan	After Fontan
Hoda	2022	2007–2021	16	–	0.3 (0.02-3) [range]	11	0.4 (0–3) [range]	2	3	5	1	5	0
Wamala	2022	2006–2017	18	35	0.02 (0.01–0.03)	11	–	18	0	0	0	0	0
Ono	2020	1999–2018	44	205	0.4 (0.3–1.6)	–	4.8 (2.3–6.7)	0	4	23	3	14	0
Alsoufi	2018	2002–2012	30	–	0.5 (0.01–4.1)	16	7.8 (3.8)	4	0	17	0	8	1
Huang	2016	2004–2013	11	37	0.02 (0.01–0.03)	–	–	11	0	0	0	0	0
Bautista-Hernandez	2014	2000–2012	35	–	1.9 (0.5–16.2)	–	3.3 (0.1–14.8)	0	0	15	4	27	7
Ruzmetov	2014	1988–2012	11	46	0.8 (0.5–2.3)	4	7.9 (6.5)	1	0	4	3	2	1
Sugiura	2014	1991–2010	26	–	0.7 (0.8)	–	4.9 (4.4)	9	0	13	3	3	0
Nakata	2010	1999–2008	12	–	–	–	3.1 (3.1)	5	2	2	1	2	0

*IQR* interquartile range; *SD* standard deviation; *TV* tricuspid valve

### Surgical Strategy

The most common surgical technique for TV repair was commissuroplasty (139/191 patients, 72.8%), followed by annuloplasty (113/191, 59.2%), neo chordae implantation (24/191, 12.6%), leaflet adaptation (20/191, 10.5%), cleft closure (16/191, 8.4%), edge-to-edge stitch (12/191, 6.3%), and other less frequent procedures (13/191, 6.8%). The study of Nakata and colleagues[37] did not report a detailed description of TV repair techniques for HLHS patients, thus was excluded from this sub-analysis. Table 2 and Supplemental Fig. 3 summarize the adopted surgical techniques in each selected manuscript.

**Table 2:** Surgical techniques for TV repair adopted in the selected studies (n = 8)

Author	Year	TV repair techniques (can be multiple in the same patient)						
		Annuloplasty	Commissuroplasty	Neo Chordae	Edge-to-Edge	Cleft Closure	Leaflet Adaptation	Other
Hoda	2022	8	9	0	3	0	0	1
Wamala	2022	8	12	8	0	0	0	0
Ono	2020	11	47	10	7	10	20	9
Alsoufi	2018	25	12	0	0	2	0	3
Huang	2016	8	3	0	0	1	0	0
Bautista-Hernandez	2014	20	45	6	0	2	0	0
Ruzmetov	2014	11	2	0	0	1	0	0
Sugiura	2014	22	9	0	2	0	0	0

*TV* tricuspid valve

### Early Outcomes and Transplant-free Survival

**Fig. 4:**
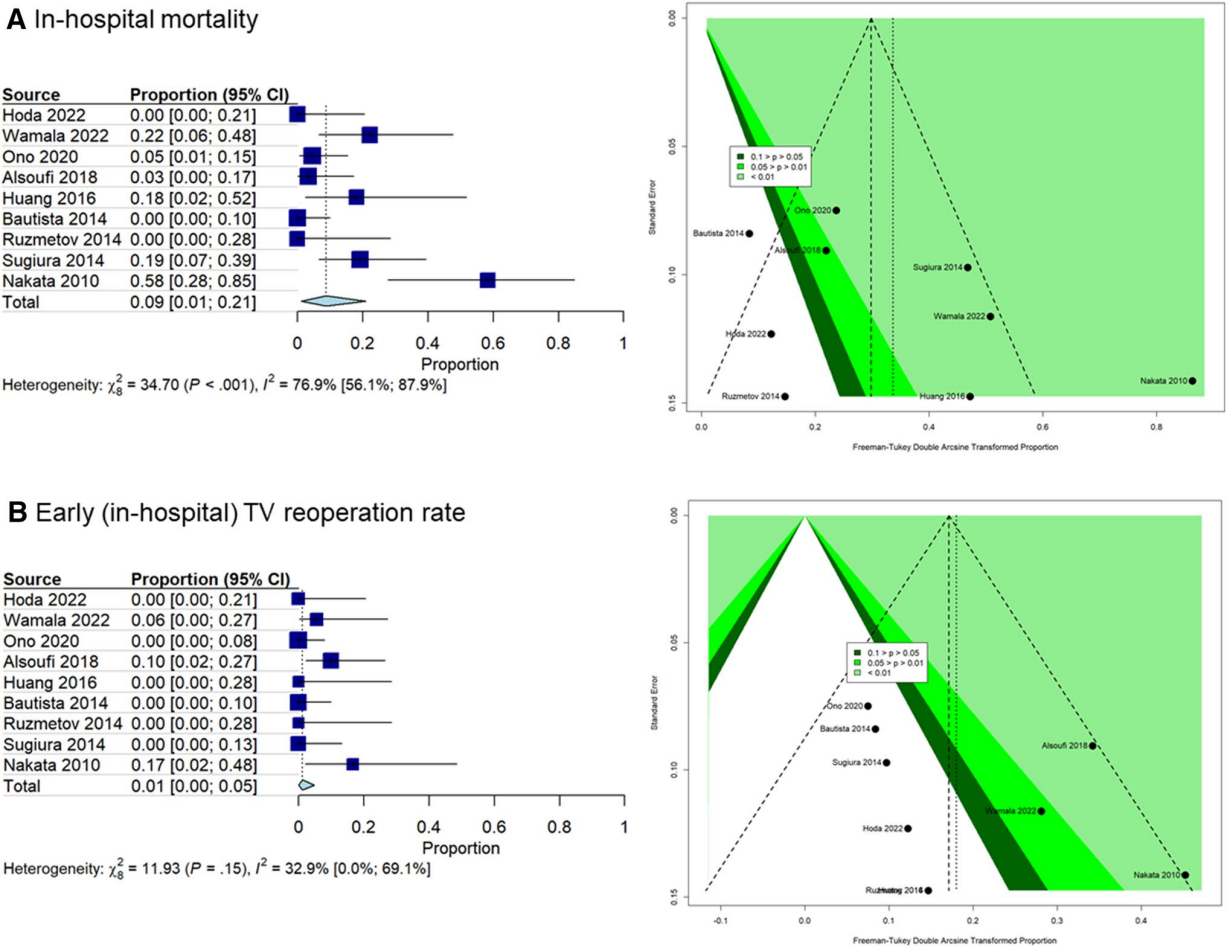
Forest plot and funnel plot of the meta-analysis of in-hospital mortality rate (panel A) and early (in-hospital) TV reoperation rate (panel B) across included studies (n=9). TV: tricuspid valve. The funnel plot contains three shaded regions. The shaded regions indicate significant effects

From pooled analysis of the included studies, in-hospital mortality after TV repair was 9% [95% CI = 1–21%; I^2^ = 76.9%, p < 0.001, Fig. 4). The rate of patients undergoing TV repair at the time of Norwood operation acted as a modifier effect on the meta-analysis (estimate 0.004 [95% CI: 0.0005–0.007] per 1% increase of Norwood rate, p = 0.024). Age at surgery (estimate − 0.16 [95% CI: − 0.33–0.01], p = 0.066), follow-up period (estimate − 0.011 [95% CI: − 0.097–0.076], p = 0.810), and cohort period (estimate − 0.024 [95% CI: − 0.065–0.017], p = 0.253) did not present a modifier effect on the analysis.

The pooled risk of early (in-hospital) TV reoperation resulted to be 1% [95% CI = 0–5%; I^2^ = 32.9%, p = 0.15, Fig. 4). None of the considered variables had a modifier effect on the meta-analysis.

**Fig. 5:**
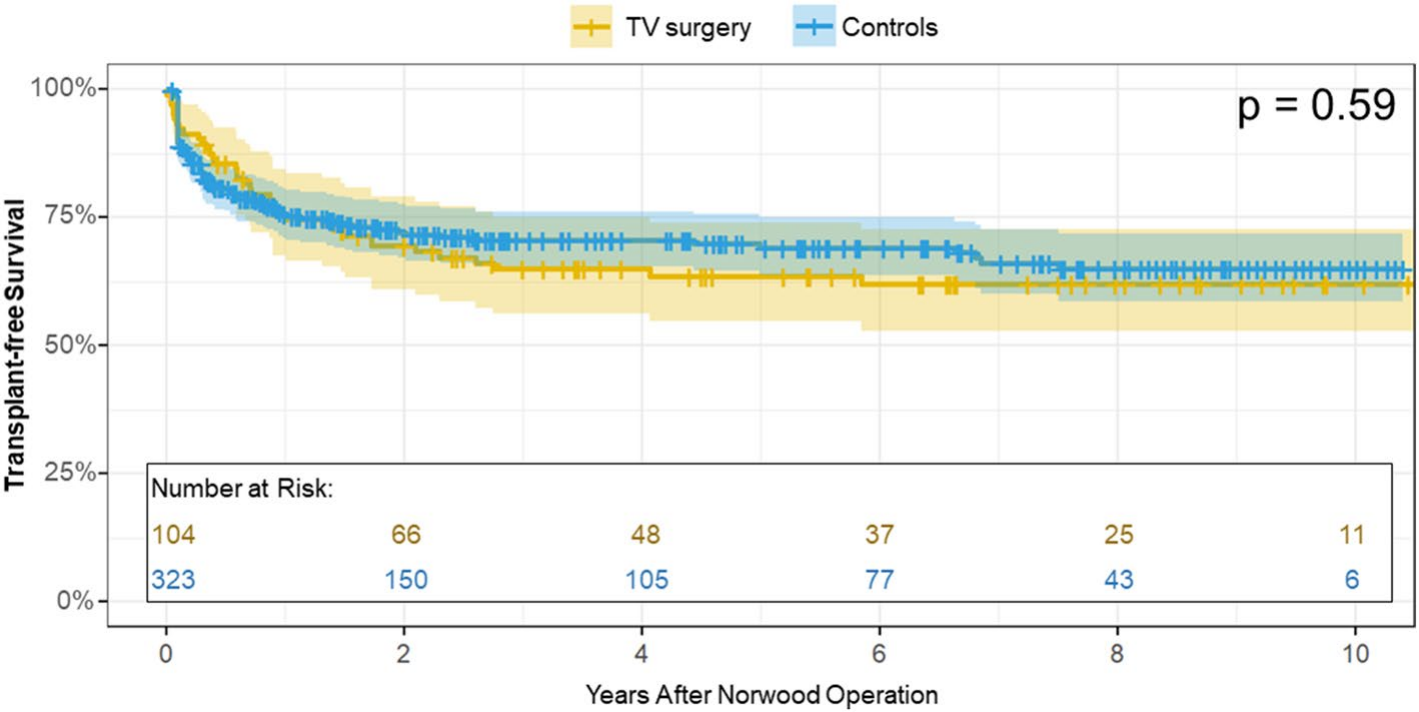
Pooled Kaplan–Meier curves of transplant-free survival of HLHS patients undergoing TV repair (n=104) vs. controls (n=323). TV: tricuspid valve

Five manuscripts [13, 15, 16, 22, 35] (for a total of 104 patients) reported Kaplan-Meier curves with a follow-up starting from the time of Norwood operation, which allowed for the reconstruction of transplant-free survival data. The meta-analysis conducted on the identified studies estimated a transplant-free survival at 1, 2, 5, and 10 years of follow-up of 75.5% [95% CI = 67.6–84.3%], 69.4% [95% CI = 60.9–79%], 63.6% [95% CI = 54.6–73.9%], and 61.9% [95% CI = 52.7–72.6%], respectively (Fig. 5). Age at surgery (HR: 0.66, 95% CI = 0.20–2.19, p = 0.497), the rate of patients undergoing TV repair at the time of Norwood operation (HR: 1.00, 95% CI = 0.99–1.01, p = 0.631), follow-up time (HR: 0.93, 95% CI = 0.77–1.14, p = 0.491), and publication year (HR: 0.95, 95% CI = 0.84–1.08, p = 0.437) did not act as effect modifiers on the meta-analysis.

Pooled transplant-free survival of patients undergoing TV repair did not differ from the one of 323 patients with HLHS without TVR used as controls [13, 15, 16, 35] (p = 0.59, Fig. 5). When selecting those patients with TVR requiring surgery (n = 84) only from studies reporting controls [13, 15, 16, 35], transplant-free survival between the two groups was still comparable (p = 0.88, Supplemental Fig. 4), with a pooled HR of mortality of 1.12 (95% CI = 0.77–1.62, p = 0.568).

### Freedom from TVR Recurrence and Freedom from Reoperation

**Fig. 6:**
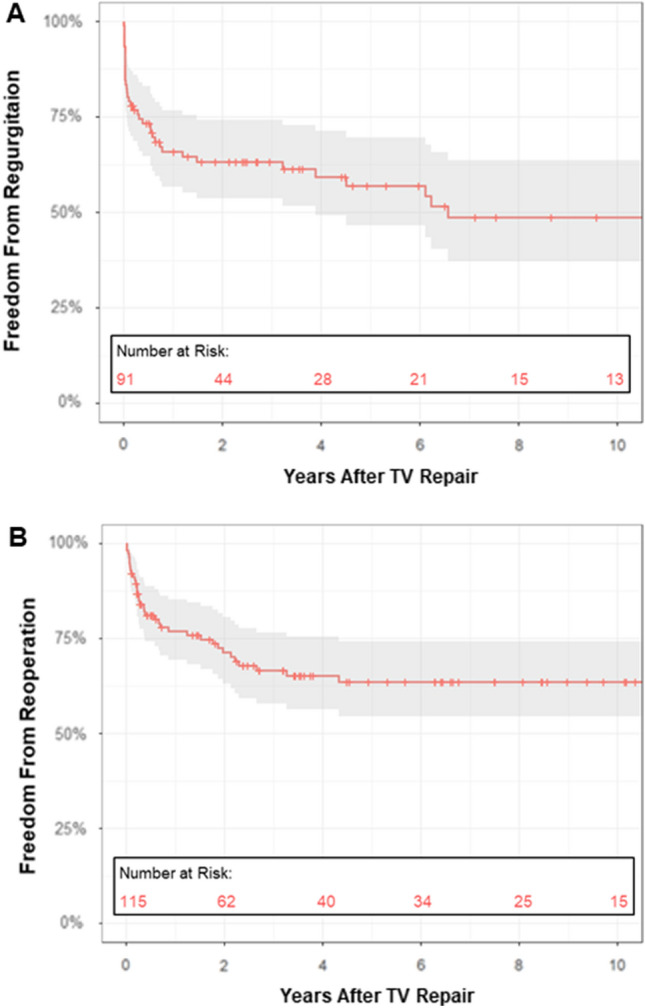
Pooled Kaplan–Meier curve of freedom from TVR recurrence (panel A). Pooled Kaplan–Meier curve of freedom from TV reoperation (panel B). TV: tricuspid valve

Four studies [13, 22, 36, 37] (including a total of 91 patients) reported estimates of freedom from recurrence of clinically significant TVR after TV repair. Pooled analysis revealed freedom from TVR recurrence at 1, 2, 5, and 10 years of follow-up of 65.9% [95% CI = 56.7–76.7%], 63.2% [95% CI = 53.8–74.3%], 57% [95% CI = 46.7–69.7%], and 48.7% [95% CI = 37.3–63.7%], respectively (Fig. 6). Age at surgery had a modifier effect on the freedom from regurgitation (HR: 0.32, 95% CI = 0.19–0.54, p < 0.001), with younger patients experiencing an increased risk of recurrence of TVR. Similarly, the rate of patients undergoing TV repair at the time of Norwood operation acted as an effect modifier (HR: 1.02, 95% CI = 1.00–1.03, p = 0.021), increasing the risk of TVR recurrence. Follow-up time (HR: 1.27, 95% CI = 0.33–4.96, p = 0.730) and publication year (HR: 1.04, 95% CI = 0.85–1.27, p = 0.708) had not a modifier effect on the meta-analysis.

Five studies [13–16, 37] (for a total of 115 patients) estimated the freedom from TV reoperation after TV repair. Pooled analysis showed freedom from TV reoperation at 1, 2, 5, and 10 years of follow-up of 77% [95% CI = 69.4–85.4%], 71.4% [95% CI = 63.1–80.7%], 63.6% [95% CI = 54.5–74.3%], and 63.6% [95% CI = 54.5–74.3%], respectively (Fig. 6). Age at surgery acted as an effect modifier (HR: 0.05, 95% CI = 0.01–0.25, p < 0.001) and younger patients displayed an increased risk of TV reoperation. The rate of patients requiring TV repair at the time of Norwood operation had a modifier effect on the freedom from TV reoperation (HR: 1.02, 95% CI = 1.01–1.02, p < 0.001). Neither follow-up time (HR: 0.85, 95% CI = 0.67–1.07, p = 0.173) nor publication year (HR: 1.08, 95% CI = 0.92–1.26, p = 0.351) acted as effect modifiers on the meta-analysis.

### Publication Bias

The funnel plots, in addition to the traditional bands to identify publication bias, contain three shaded regions; these regions identified statistically significant effects for a significance level between 0.1 and 0.05, 0.05 and 0.01, and < 0.01. Concerning the in-hospital mortality outcome only two studies fail outside the funnel plot bounds indicating a controlled publication bias; the only reporting a significant effect despite the high standard error is Nakata et al [37].

The studies reporting the early (in-hospital) TV reoperation rate outcome fall all inside the funnel plot bounds indicating a substantial absence of publication bias.

## Discussion

Up to 1/4 of children affected by HLHS are projected to develop clinically significant TVR necessitating surgical repair during their single-ventricle palliation course [8, 9, 38]. Given the intrinsic pathophysiological relationship between TVR and RV myocardial remodeling triggered by its inclusion in the systemic circulation (Fig. 1), the role of surgical TV repair in interrupting this vicious cycle and positively modifying the long-term prognosis of HLHS patients is still undefined. We specifically addressed this topic by performing a meta-analysis of scientific literature which revealed that patients undergoing TV repair display comparable transplant-free survival to HLHS peers without clinically significant TVR. On the other hand, the durability of surgical repair seems to be limited and a significant quote of patients will necessitate more than one surgical procedure on the TV.

The loss of systemic TV competence represents a bad prognostic factor at every stage of univentricular palliation, impacting interstage I mortality [9], interstage II mortality [8], and Fontan completion outcomes [39]. In the multicenter prospective cohort of the Single Ventricle Reconstruction Trial, 11/549 patients required TV repair at the time of Norwood procedure [40], 44/393 at stage II operation [8], and 29/327 at Fontan [39]. However, the outcomes of TV surgery have not been specifically discussed by the investigators. More recently, in a sub-analysis of the Australia and New Zealand Fontan Registry, patients with HLHS exhibited poor long-term freedom from atrio-ventricular valve failure, which was demonstrated to be associated with RV contractile dysfunction and failure of Fontan circulation [17]. Interestingly, the observed inferior prognosis of Fontan patients requiring atrio-ventricular valve surgery resulted to be mainly driven by a disproportionate effect that atrio-ventricular valve regurgitation displayed in the RV-dominant population only [10]. These findings suggest that a successful TV surgery may redefine the natural history of TVR in patients with RV dominance. Unfortunately, HLHS patients represented only 35% (205/581) of the original right-dominant cohort, with only 42/205 cases requiring TV repair [10], thus limiting the applicability of findings.

By performing a systematic review of scientific literature, we sought to define if TV surgery can effectively modify the prognosis of HLHS patients with TVR. Keeping in mind the intrinsic limitations of a meta-analysis of retrospective observational studies, in this delicate cohort we observed a pooled transplant-free survival at 1, 5, and 10 years of follow-up of 75.5% [95% CI = 67.6–84.3%], 63.6% [95% CI = 54.6–73.9%], and 61.9% [95% CI = 52.7–72.6%], respectively, which parallels the prognosis of the general population of HLHS from large multicenter studies [38, 41]. In fact, both our overall cohort and the cases selected only from studies reporting controls (Supplemental Fig. 4) displayed a similar prognosis to HLHS peers without TVR (p = 0.59 and p = 0.88, respectively). Our results may imply that TV repair, together with the improved pre- and post-operative medical management [42, 43], can restore the original prognosis of HLHS patients with TVR, counteracting the deleterious effect that untreated TVR has on the patient’s survival.

Insights from 3-dimensional echocardiography have revealed that the mechanisms of TVR in HLHS entail flattening and dilatation of TV annulus, together with leaflet prolapse and tethering [19, 20]. Surgical repair is proven to effectively address annular enlargement, commissural regurgitant jets, and posterior leaflet prolapse [19]. However, septal leaflet tethering, which is directly related to RV cavity dilatation and contractility [20], is poorly modified by surgical efforts [19] and represents a risk factor for failure of TV repair [19]. The complex interdependence of TVR and RV myocardium might account for the high rates of TVR recurrence that we estimated through our meta-analysis. Almost half of the patients will experience a relapse of significant (≥ moderate) TVR at a medium follow-up (Fig. 6), translating into the need for a second surgical repair in most cases. Meta-regression analysis confirmed that a younger age at TV repair and TV repair occurring at the time of Norwood operation can augment the risk of TVR recurrence and the reoperation rate. We speculate that the higher technical complexity of TV surgery in smaller patients and, possibly, the presence of TV structural abnormalities or more compromised RV function may be the major drivers of the increased hazards in this subgroup of patients [36].

Our results should be carefully interpreted in light of the relatively short mean follow-up times of included studies (Table 1). Although a favorable RV remodeling process has been documented early after TV repair in HLHS [23, 36], which may sustain the positive effect of surgery on the patient’s survival, the very-long term fate of TV in HLHS is still to define. We may hypothesize that the observed high rates of TV recurrence and the need for TV reoperation indicate a strong interdependence of TVR and myocardial performance, which has been recognized to progressively decline when the RV is adopted as the systemic pumping chamber [6, 10, 44]. In this view, a later deflection of survival estimates from HLHS peers without TVR cannot be excluded, imposing strict and structured clinical surveillance even in patients in whom a successful TV repair has been achieved.

### Limitations

Conducting a meta-analysis of observational studies possesses intrinsic limitations that our study has to account for. Unfortunately, the only large randomized clinical trial enrolling HLHS patients (the Single Ventricle Reconstruction Trial) has not specifically addressed the effects of TV repair on outcomes, thus it could not be included in our meta-analysis. We hope that our work could stimulate novel analysis of this precious source of clinical data on HLHS. Comparing outcomes of cases vs. controls that have been enrolled from different populations may generate a selection bias. However, when we compared cases vs. controls extracted from the same studies we did not observe a statistically significant modification of our results (see Supplemental Fig. 4). In order to define the source of heterogeneity among studies we investigated the modifier effect of a relatively small number of variables and we cannot exclude that other parameters may contribute to the studies heterogeneity. The individual patient data reconstruction of the Kaplan–Meier curves allows the characterization of long-term endpoints for large composed cohorts of patients. However, this pooled estimate does not account for the patient’s specific characteristics and possible confounding factors affecting the outcome. Finally, the relatively short mean follow-up times of included studies don’t allow reliable inferences on the very long-term fate of TV competence and patients’ survival after TV repair.

## Conclusions

At a medium-term follow-up, TV repair can effectively modify the prognosis of patients with HLHS and loss of systemic TV competence, reestablishing a comparable transplant-free survival to HLHS peers without TVR. However, the durability of surgery seems to be time-dependent and a significant quota of patients will experience TVR recurrence, requiring more than one surgical procedure on the TV. The intrinsic relationship between TV competence and RV remodeling dictates careful and pro-active surveillance of this delicate population.

## Supplementary Information

Below is the link to the electronic supplementary material.

Results of quality assessment using AXIS tool for each study included in the meta-analysis. Supplementary material 1 (TIF 215.0 kb)

Timing of TV repair during single-ventricle palliation course in the included studies (n=9). TV: tricuspid valve. Supplementary material 2 (TIF 32.4 kb)

Techniques for TV repair reported in the selected studies (n=8). TV: tricuspid valve. Supplementary material 3 (TIF 36.8 kb)

Pooled Kaplan–Meier curves of transplant-free survival of patients requiring TV repair (n=84) selected only from studies reporting controls (n=323), with estimated HR across studies. CI: confidence interval; HR: hazard ratio; TV: tricuspid valve. Supplementary material 4 (TIF 149.4 kb)

## Data Availability

Data available on request to the corresponding author.

## Abbreviations

CI: confidence interval
HLHS: hypoplastic left heart syndrome
HR: hazard ratio
IQR: interquartile range
LV: left ventricle
RV: right ventricle
SD: standard deviation
TV: tricuspid valve
TVR: tricuspid valve regurgitation
