# Supplementary figures and images for: Tricuspid Valve Repair Can Restore the Prognosis of Patients with Hypoplastic Left Heart Syndrome and Tricuspid Valve Regurgitation: A Meta-analysis
Source: Pediatr Cardiol. 2023 Aug 9;45(8):1702–12. doi: 10.1007/s00246-023-03256-0 (PMC11442528; doi:10.1007/s00246-023-03256-0)

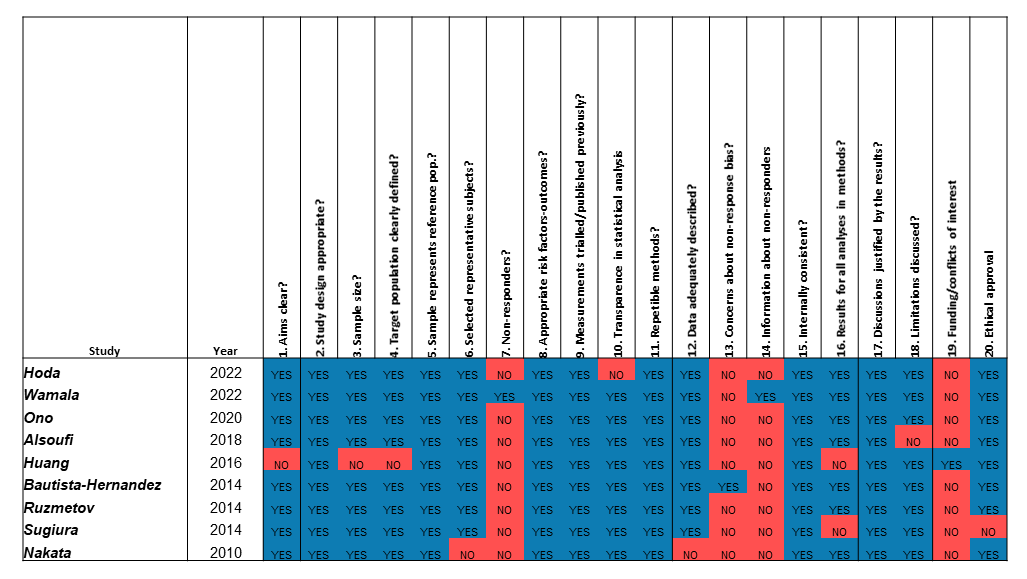

Supplement: Supplementary file 1 — Results of quality assessment using AXIS tool for each study included in the meta-analysis. Supplementary material 1 (TIF 215.0 kb) [file 246_2023_3256_MOESM1_ESM.tif]

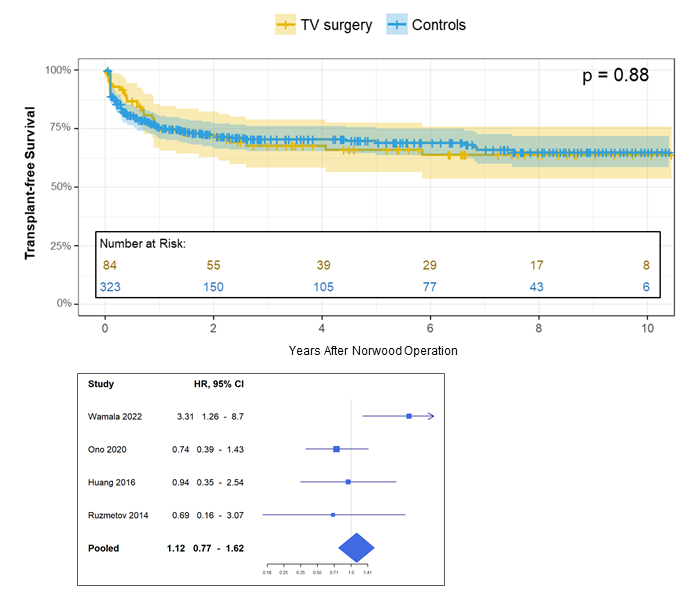

Supplement: Supplementary file 4 — Pooled Kaplan–Meier curves of transplant-free survival of patients requiring TV repair (n=84) selected only from studies reporting controls (n=323), with estimated HR across studies. CI: confidence interval; HR: hazard ratio; TV: tricuspid valve. Supplementary material 4 (TIF 149.4 kb) [file 246_2023_3256_MOESM4_ESM.tif]
